# An Integrated Approach Based on Clinical Data Combined with Metabolites and Biomarkers for the Assessment of Post-Operative Complications after Cardiac Surgery

**DOI:** 10.3390/jcm13175054

**Published:** 2024-08-26

**Authors:** Peter Meinarovich, Alisa Pautova, Evgenii Zuev, Ekaterina Sorokina, Ekaterina Chernevskaya, Natalia Beloborodova

**Affiliations:** Federal Research and Clinical Center of Intensive Care Medicine and Rehabilitology, 25-2 Petrovka Str., 107031 Moscow, Russia; pmejnarovic@gmail.com (P.M.); zuev17ev@gmail.com (E.Z.); esorokina@fnkcrr.ru (E.S.); echernevskaya@fnkcrr.ru (E.C.); nvbeloborodova@yandex.ru (N.B.)

**Keywords:** aromatic microbial metabolites, sepsis-associated metabolites, 4-hydroxyphenyllactic acid, prognostic models, SOFA

## Abstract

**Background:** Early diagnosis of post-operative complications is an urgent task, allowing timely prescribing of appropriate therapy and reducing the cost of patient treatment. The purpose of this study was to determine whether an integrated approach based on clinical data, along with metabolites and biomarkers, had greater predictive value than the models built on fewer data in the early diagnosis of post-operative complications after cardiac surgery. **Methods**: The study included patients (*n* = 62) admitted for planned cardiac surgery (coronary artery bypass grafting with cardiopulmonary bypass) with (*n* = 26) or without (*n* = 36) post-operative complications. Clinical and laboratory data on the first day after surgery were analyzed. Additionally, patients’ blood samples were collected before and on the first day after surgery to determine biomarkers and metabolites. **Results**: Multivariate PLS-DA models, predicting the presence or absence of post-operative complications, were built using clinical data, concentrations of metabolites and biomarkers, and the entire data set (ROC-AUC = 0.80, 0.71, and 0.85, respectively). For comparison, we built univariate models using the EuroScore2 and SOFA scales, concentrations of lactate, the dynamic changes of 4-hydroxyphenyllactic acid, and the sum of three sepsis-associated metabolites (ROC-AUC = 0.54, 0.79, 0.62, 0.58, and 0.70, respectively). **Conclusions:** The proposed complex model using the entire dataset had the best characteristics, which confirms the expediency of searching for new predictive models based on a variety of factors.

## 1. Introduction

The incidence of complications after cardiac surgery has decreased due to the development of new technologies and treatment methods, as well as improved qualifications of surgeons. However, the development of various complications still remains a serious problem that can lead to increasing expense for treatment, mortality, or deterioration in the patient’s quality of life after surgery [1]. The Society of Thoracic Surgeons has identified five major post-operative complications: stroke, renal failure, prolonged intubation, unplanned reoperation, and deep sternal wound infection. The frequency of complications may vary depending on the type of operation, the patient’s condition before surgery, and the professionalism of the operating team of cardiac surgeons [2]. To reduce the risks of post-operative complications, various measures are used, such as careful planning of the operation, preparing the patient before the operation, and strict monitoring in the post-operative period. 

The use of biomarkers is a powerful tool that can improve the quality of the diagnostics and treatment of patients, help to understand the patient’s condition, and promptly identify possible complications. These biomarkers are lactate [3], procalcitonin (PCT), interleukin-6 (IL6), N-terminal pro B-type natriuretic peptide (NT-proBNP), protein S100 [4], and high-sensitivity troponin T (HST-T) [5]. They have different sensitivity and specificity for the prognosis of the post-cardiosurgical complications, however, none of them is an excellent biomarker with 100% sensitivity and specificity.

Various metabolites have increasingly been used in clinical practice and actively studied for early diagnosis [6,7,8,9,10]. The value of metabolites lies in their ability to mark not only changes in the human body. The gut microbiota is known to influence host physiology, including through the production of multiple metabolites [11]. There is a concept pertaining to the relationship between the gut microbiota and the heart, the so-called gut–heart axis, which has led to novel treatment and prevention strategies by studying and targeting the composition of the gut microbiome and its metabolites [4,12,13]. Most metabolomic studies in cardiac surgery are usually related to the development of certain complications, such as acute kidney injury [7,14,15] and post-operative delirium [8,16]. However, our hypothesis is that an altered microbiome both before and in the early post-operative period and, as a consequence, its altered metabolic function, may be the cause of the development of any type of post-operative complication.

In one of our recent single-center studies on aortic prosthetics, aromatic microbial metabolites were increased in the early post-operative period (6 h after the end of the surgery) in the group of patients with all types of post-operative complications (*n* = 43) and, in particular, in patients with infectious complications (*n* = 26) [17]. In our other single-center pilot study on patients undergoing cardiac surgery (*n* = 24), those who developed infectious complications (*n* = 12) in the early post-operative period had a distinct gut microbiota taxonomy, with a predominance of potentially pathogenic species, even before the surgery [18]. In the current study, we decided to further analyze certain metabolites in all cardiac surgery patients from this study, as well as examine known biomarkers to try to predict complications that may occur. Therefore, the aim of this study was to identify the value of metabolite and biomarker monitoring for predicting post-operative complications in cardiac surgery in comparison with clinical data.

## 2. Materials and Methods

### 2.1. Study Design

This study was performed in the N. Pirogov National Medical Surgical Center, Moscow, Russian Federation. The local Ethics Committee approved the study (no. 04 from 22 May 2018), which was conducted in accordance with the ethical standards of the Declaration of Helsinki. Informed consent for participation in this study was also obtained from each patient or his/her legal representative. 

The criteria for inclusion in the study were the following: the patient was over 18 years old, had planned cardiovascular surgery that corresponded to one of the following categories of cardiac surgery— coronary artery bypass grafting (CABG) or combination of CABG with other types of cardiac surgery. Also, serum samples should be collected before the surgery (point 0) or within a day following the surgery (point 1). Additionally, the patient gave informed consent to participate in the study. 

Patients with the following criteria were not considered in this study: the presence of active infectious endocarditis, emergency surgery, previous bacterial infectious diseases in the past three months, antibiotic use within the past three months, or refusal to participate in the study.

Exclusion criteria included off-pump CABG and the absence of one of the collected samples (point 0 or point 1). All patients were divided into two groups retrospectively: with post-surgical complications (*n* = 26) and without post-surgical complications (*n* = 36). Complications included post-operative bleeding (*n* = 9, 15%), respiratory disorders (*n* = 7, 11%), atrial fibrillation (*n* = 5, 8%), myocardial ischemia (*n* = 5, 8%), and delirium (*n* = 2, 3%). Some patients had multiple organ failure (*n* = 3, 5%). Demographic and peri-operative information, comorbidity, scales, and laboratory parameters were retrospectively analyzed from medical documentation.

**Figure 1:**
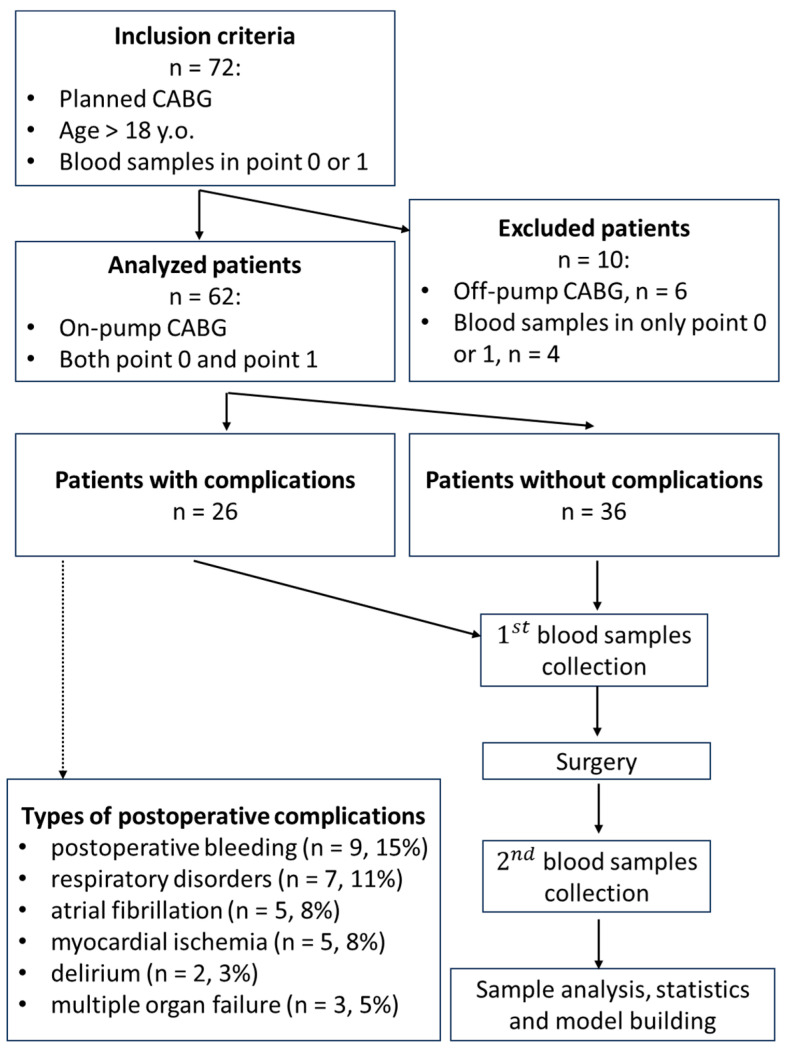
Study design summary (CABG—coronary artery bypass grafting).

Initially, 72 patients were included in the study, however, only 62 of them met the necessary criteria for inclusion, i.e., had a cardiopulmonary bypass during the cardiac surgery and had serum samples in both points 0 and 1. Patients (*n* = 62, including 44 males and 18 females) had different types of planned cardiovascular surgery. All patients had coronary artery bypass grafting—CABG (*n* = 62, 100%); some patients had combined operations—CABG and plastics of aortic or mitral valve (PAMV) (*n* = 12, 19%), CABG, and post-infarction left ventricular aneurysm resection (*n* = 2, 3%). All surgeries were performed with a cardiopulmonary bypass. Some patients had the following concomitant diseases: chronic heart failure (*n* = 54, 87%), chronic gastritis (*n* = 45, 73%), hypertension (*n* = 44, 71%), diabetes (*n* = 10, 16%), atrial fibrillation (*n* = 7, 11%), obesity (*n* = 5, 8%), gastric ulcers (*n* = 4, 7%), and chronic pancreatitis (*n* = 4, 7%). The median (interquartile range 25, 75%) age was 62 (57, 68) years, ejection fraction (EF) was 60 (49, 64) %. All patients used peri-operative antibiotic prophylaxis, using 2 g of cefazolin three times within 24 h. Summary information concerning the included and excluded patients is presented in Figure 1.

### 2.2. Blood Sample Collection

The collection of patients’ blood samples was performed before the surgery (point 0) and within a day after the surgery (point 1). There were also 48 blood samples from healthy donors (*n* = 48), which were used to reveal reference values for aromatic and dicarboxylic acid concentrations [17]. Serum samples were obtained by blood centrifuging at 1500× *g* for 10 min on the same day. The total number of serum samples (*n* = 172) included 124 from patients and 48 from healthy volunteers. 

Data on the following parameters were taken from the medical documentation: EF% before surgery, hemoglobin (Hb), leucocytes, the highest value of lactate for the first day (lactate), pH max, pH min, infusion volume, on-pump time, number of erythrocytes transfusions, and total blood loss. Additionally, some specific analyses were conducted in the Federal Research and Clinical Center of Intensive Care Medicine and Rehabilitology in Moscow, Russia. Gas chromatography-mass spectrometry (GC-MS) analyses using a Trace GC 1310 gas chromatograph and ISQ LT mass spectrometer from the Thermo Electron Corporation (Santa Clara, CA, USA) were conducted to measure the concentration of various aromatic and dicarboxylic acids (benzoic acid—BA, phenylacetic acid—PhAA, phenylpropionic acid—PhPA, phenyllactic acid—PhLA, 4-hydroxybenzoic acid—p-HBA, 4-hydroxyphenylacetic acid—p-HPhAA, 4-hydroxyphenylpropionic acid—p-HPhPA, homovanillic acid—HVA, 4-hydroxyphenyllactic acid—p-HPhLA, succinic acid—SA, and fumaric acid—FA). The limit of quantitation for all acids was 0.5 µmol/L with a relative standard deviation of 10–30%, and the calibration curve was linear for all metabolites within the clinically significant concentration range (0.5–15 µmol/L). The sample preparation for the analytes and the details on the GC-MS analysis were previously described [19]. Several biomarkers were measured (IL6, PCT, NT-proBNP, protein S100, and HST-T) by electrochemiluminescence (Cobas e411, Roche, Basel, Switzerland).

### 2.3. Statistical Analysis

**Figure 2:**
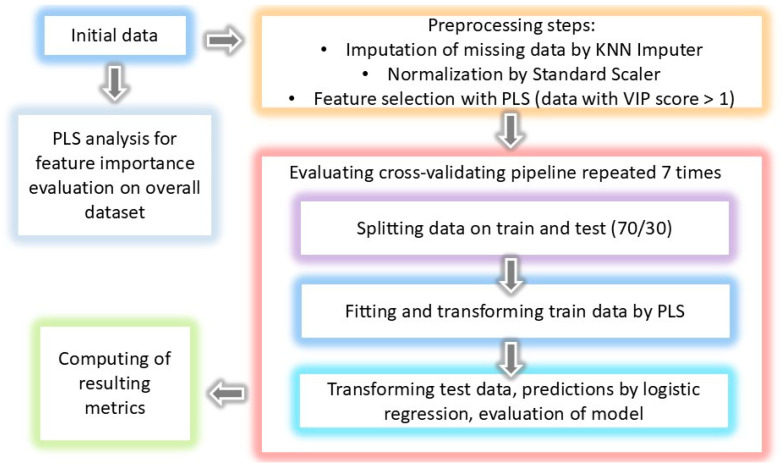
Resulting pipeline for building multivariate predictive models.

The Shapiro–Wilk test was used to assess the normality of the data distribution and, as a result, there were no data distributed normally. Continuous variables were described in tables using median and interquartile ranges; categorial variables were described using a number of cases and percentages. The Mann–Whitney U-test (two-sided) and Chi-square test were used for primary descriptive statistics in Section 3.1, and *p*-value < 0.05 was chosen as significant. All statistical tests were performed with Scipy 1.14.1, Python. The proportion of missing data was less than 5%, primarily in biomarkers. A k-nearest neighbors (KNN) imputer was used to fill in missing values. To achieve dimensional reduction for visualization and machine learning, partial least squares (PLS) regression was used (Section 3.2). Variable importance in projection (VIP) scores were computed to evaluate the importance of variables. Three multivariate and five univariate models were built. For multivariate models, initial lists of variables were used for fitting PLS data with important features (VIP score > 1) then transformed by PLS and used for the fitting of logistic regression and binary classification (PLS-DA). The schematic pipeline is shown in Figure 2. For univariate models, receiver operating characteristic (ROC) analysis was performed. All models were performed with Sklearn 1.3.1 (Python) and metrics (area under the ROC curve (ROC-AUC), sensitivity, specificity) with 95% confidence intervals (95% CI) computed and evaluated with k-fold cross-validation (k = 7).

## 3. Results

### 3.1. Comparison of Uncomplicated and Complicated Patient Groups

Table 1 describes the medical and demographic characteristics, intraoperative parameters, and the serum concentrations of metabolites and biomarkers in all patients (*n* = 62), which were compared to the corresponding reference values or donors’ concentrations for metabolites, and the differences between patients without (*n* = 36) and with (*n* = 26) complications before (point 0) and within a day after the surgery (point 1). 

In point 0 there were patients with the following parameters that were beyond the reference values: hemoglobin (*n* = 23, 37%), leucocytes (*n* = 58, 94%), lactate (*n* = 62, 100%), pH (*n* = 39, 63%), IL6 (*n* = 33, 53%), NT-proBNP (*n* = 50, 81%,), S100 (*n* = 8, 13%), and HST-T (*n* = 48, 77%). According to the criterion that 95% CI of the parameter should not contain the minimum or maximum level of the corresponding reference range, the following parameters should be considered as significantly beyond the reference range: leukocytes in all patients—95% CI (11.3 – 20.8) × 10^9^ (beyond reference range (4–9) × 10^9^) and lactate in all patients—95% CI 2.82 – 7.51 mmol/L (higher than the maximum reference level of 2 mmol/L).

Usage of vasopressors (*p* = 0.02), time of mechanic ventilation (*p* < 0.001), infusion volume (*p* < 0.001), on-pump time (*p* < 0.001), number of erythrocyte transfusions (*p* = 0.03), blood loss on the 1st day (*p* = 0.04), Sepsis-related Organ Failure Assessment (SOFA) scores (*p* < 0.001), and HST-T after the surgery (*p* = 0.03) were significantly higher in patients with complications compared to uncomplicated ones.

In serum samples of donors and all patients before the surgery (in point 0), differences in concentrations of BA (*p* < 0.001), PhAA (*p* < 0.001), p-HBA (*p* = 0.03), p-HPhAA (*p* < 0.001), sum of sepsis-associated PhLA, p-HPhAA and p-HPhLA—Σ3AMM (*p* = 0.04), SA (*p* < 0.001), and FA (*p* < 0.001) were statistically significant. Within a day after the surgery, all metabolites differed statistically in all donors and patients.

The dynamics of the metabolites and biomarkers were evaluated as the difference (Δ) between their levels in points 1 and 0 (1–0). The differences in the level of Σ3AMM (*p* = 0.03), PCT (*p* = 0.01), NT-proBNP (*p* = 0.01), and HST-T (*p* = 0.01) were statistically significant.

### 3.2. Models for the Prognosis of the Post-Operative Complications

**Figure 3:**
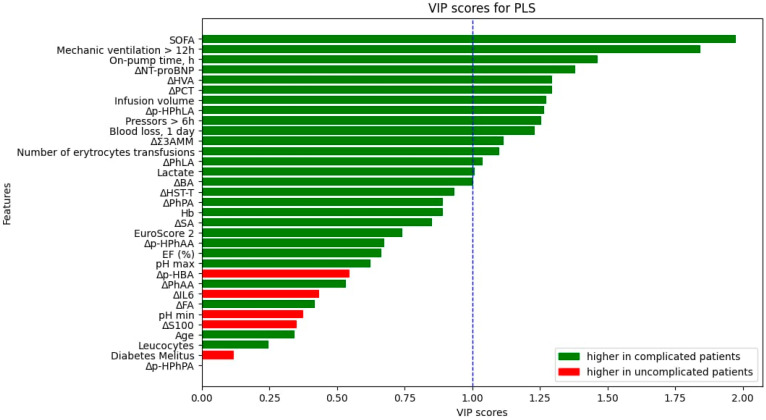
VIP scores for PLS analysis. The blue line marks the threshold of importance (VIP score > 1.0).

Data that were collected before and within a day after the surgery were used to fit models to predict if a patient would have complications or not. PLS analysis was performed for the importance evaluation of all parameters included in the model (Figure 3). The threshold value for VIP scores was chosen as 1. SOFA and time of mechanic ventilation > 12 h took leading positions in the VIP scores rating, and the clinical parameters higher than the threshold were on-pump time, infusion volume, total blood loss, number of erythrocytes transfusions, and lactate. Differences in certain metabolites and biomarkers (NT-proBNP, HVA, PCT, p-HPhLA, Σ3AMM, PhLA, and BA) were more than the chosen threshold.

**Figure 4:**
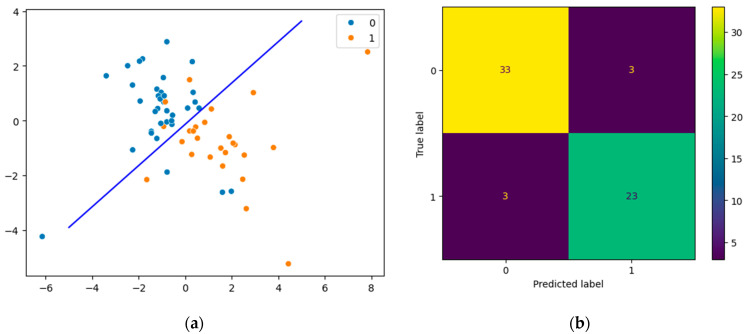
Classification results of the final model fit on all data: scatter plot (**a**) and confusion matrix (**b**). Blue points (0) correspond to patients without complications (*n* = 36), orange points (1) correspond to patients with complications (*n* = 26) in (**a**).

The resulting pipeline included transforming data with PLS and then predicting the development of complications by the logistic regression. Computed coordinates that are explained as a scatter plot (Figure 4a) show linear discrimination between groups. A confusion matrix shows the quality of classification of the final model on all data (Figure 4b). Three samples were misclassified in the uncomplicated group of patients (false negative samples) and three samples were misclassified in the complicated group of patients (false positive samples).

**Figure 5:**
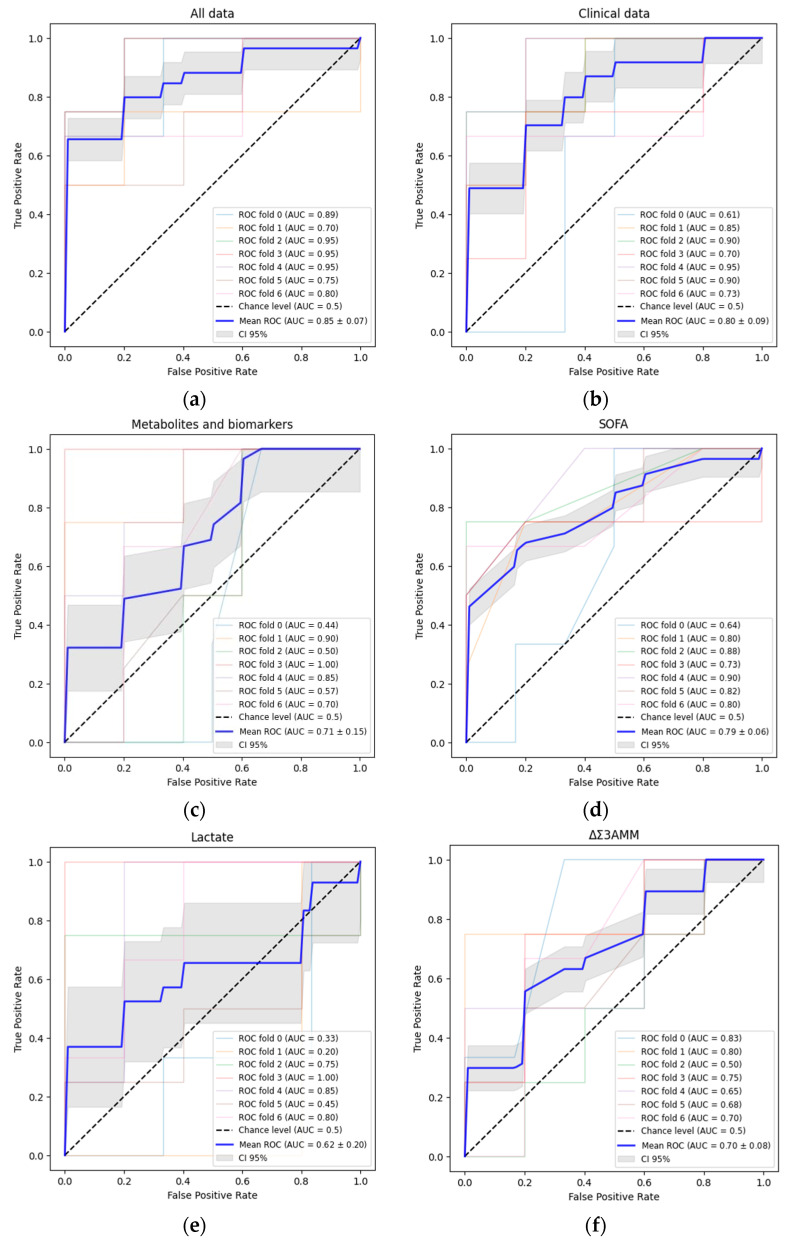

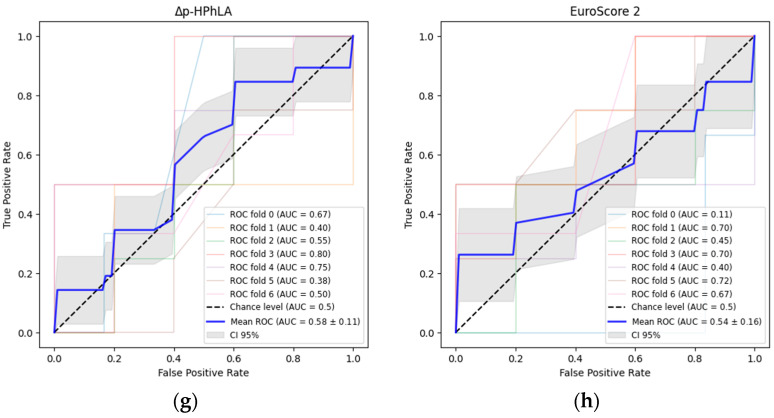
ROC curves for three multivariate models based on all data (**a**), clinical data (**b**), metabolites and biomarkers (**c**); and five univariate models based on SOFA (**d**), lactate (**e**), ΔΣ3AMM (**f**), Δp-HPhLA (**g**), and EuroScore2 (**h**). Blue lines explain means of true positive rate/false positive rate co-ordinates. Other lines show different cross-validation splits. Grey zone demonstrates 95% CI.

Multivariate PLS-DA models were constructed. We built model-fits on all data (ROC-AUC = 0.85), on only clinical data (ROC-AUC = 0.80), and on only metabolites and biomarkers (ROC-AUC = 0.71). We compared our models with univariate models built on SOFA (ROC-AUC = 0.79) and EuroScore 2 (ROC-AUC = 0.54) scores, lactate (ROC-AUC = 0.62), Δp-HPhLA (ROC-AUC = 0.58), and ΔΣ3AMM (ROC-AUC = 0.70). All data obtained were accumulated in Table 2 and Figure 5.

## 4. Discussion

Cardiac surgery remains one of the types of operation after which complications can arise, despite the development of medicine and the improvement in the quality of medical care provided. Early prediction of post-operative complications is crucial. In the early post-operative period, either medical prophylaxis or simply stricter monitoring can be performed to effectively avoid undesirable consequences of invasive treatment [16]. This was the main reason why we focused on data that were available in the pre-operative and early peri-operative periods in the current study. 

Researchers have identified various risk factors for the development of post-operative complications. They can be divided into three groups: patient characteristics (female sex, obesity, smoking), clinician characteristics (volume of infusion, duration of surgery, doses of assigned medications), and post-operative factors (clinical decisions and nursing) [2,20]. The use of cardiopulmonary bypass in cardiac surgery is considered to be one of the main risk factors that disrupts metabolic pathways, mainly due to the activation of oxidative stress [21]. This fact served as the basis for including in our study only those patients who had cardiac surgery with a cardiopulmonary bypass, to search for parameters that could distinguish patients with and without complications. In our study, clinical parameters that significantly differ in patients with and without complications were infusion volume, on-pump time, SOFA, number of erythrocytes transfusion, and blood loss in the 1st day (Table 1). Data on on-pump time correlated with another study where this parameter was also higher in the complicated group of patients [22].

Biomarkers are widely used in cardiac surgery. All of them generally explain one or a few types of complication. NT-proBNP marks atrial wall distraction [23], cardiac troponins—biomarkers of ischemic processes in cardiac tissue [5,24], PCT and IL6—markers of inflammation and infectious processes [25], lactate—a marker of tissue hypoxia [26] in prediction of post-operative complications after cardiac surgery. Inflammatory biomarkers IL6 and PCT are not always specific for assessing the severity of the infectious process in cardiac surgery patients. Cardiac surgery causes an increase in the PCT level, even in the absence of complications, but its level usually does not exceed 5 ng/mL. Patients with a complicated post-operative course, with infection or sepsis, showed higher PCT and IL6 levels than patients with an uncomplicated course [27,28,29]. Endogenous intoxication due to infection can worsen the function of the heart, which can manifest itself both clinically and in a laboratory. NT-proBNP is used in the diagnosis of heart failure. Its values always increase during the first few days after open heart surgery, with a further gradual decrease if there are no complications. Typically, NT-proBNP values are higher in more severe patients receiving inotropic therapy. Another promising biomarker to evaluate post-operative complications is HST-T, the release of which should be expected after all CABG procedures. It depends on the procedure, the nature of the cardioplegia, and many other factors. The peak HST-T concentration usually occurs within 24 to 48 h after the operation [30]. In this study, the level of HST-T in the complicated group was significantly higher than in the group of patients without complications. This can be explained by the fact that infectious complications developed more in those patients who had primary cardiac complications in the intraoperative or early post-operative periods: myocardial infarction, myocardial injury, and severe heart failure. Patients with primary cardiac complications have a greater risk of bacterial infection [31]. However, our findings correlate with data from a study that included 1318 patients after CABG surgery, with a peak HST-T level greater than 400 ng/L, measured within 24 h, and associated with a major adverse cardiac or cerebrovascular event, 30-day mortality, myocardial infection, and ICU stay>48 h [32].

Various studies were conducted to reveal metabolic changes after cardiac surgery [7,8,10]. In our previous study, higher serum concentrations of metabolites of phenylalanine and tyrosine in the complicated group of cardiac surgery patients were detected [17]. In the current study, among metabolites and biomarkers, only HST-T after the surgery differed significantly in two groups of patients, but differences between metabolites and biomarkers (point 1–point 0) were statistically significant among a few characteristics (ΔΣ3AMM, ΔPCT, ΔNT-pro-BNP, ΔHST-T). These facts show that dynamic changes are important for the prediction of post-operative complications. In our previous study, additional statistically significant differences in ∆p-HPhAA and ∆p-HPhLA were obtained [17], which we did not observe in the current study, but we obtained statistically significant differences in the dynamics of the sum of p-HPhAA and ∆p-HPhLA, together with PhLA—ΔΣ3AMM. These two studies repeatedly demonstrated the altered metabolism of phenylalanine and tyrosine in high-risk patients, with a predominance of the sepsis-associated metabolites of microbial origin [33].

Machine learning is a powerful tool that can be used for the prediction of post-operative complications. In cardiac surgery, it has been successfully used for the prognostication of post-operative atrial fibrillation [12] and acute kidney injury [34]. Although some studies have described the results as applying predictive models built only on clinical data, they have demonstrated different predictive ability. Zhang et al. reported predictive models with ROC-AUC = 0.94 for XGBoost and ROC-AUC = 0.75 for support vector machines built on records of monitoring during off-pump CABG [35]. Li et al. demonstrated ROC-AUC for different models lower than 0.70 [22]. Despite the relatively good predictive ability of clinical data, prognostic models based on metabolomic data have shown growing popularity in diagnostics because of their potential. In particular, in patients with aortic dissection, there have been results showing the marking of impaired metabolic pathways, and suggesting potential biomarkers for different conditions and diseases [10]. There have also been attempts to use metabolites and biomarkers with clinical data in machine learning for the prediction of post-operative delirium and acute kidney injury [16,36]. Our PLS-DA models fitted on metabolites and biomarkers demonstrated moderate predictive value with ROC-AUC = 0.71 and sensitivity/specificity 0.72/0.47. The model fitted only on clinical data demonstrated better predictive value with ROC-AUC = 0.80 and sensitivity/specificity 0.66/0.78. Finally, the best results were obtained for the model with the entire set of data—ROC-AUC = 0.85 and sensitivity/specificity of 0.81/0.79. This fact gives us the opportunity to suppose that the use of metabolites and biomarkers in predictive models in cardiac surgery may improve their sensitivity and specificity for recognizing post-surgical complications. 

As additional data and for comparison, we built a series of univariate models. In the current study, EuroScore2 scale performed poorly on patient group discrimination (ROC-AUC = 0.54 and sensitivity/specificity of 0.49/0.63). As noted, baseline microbiome parameters (microbial metabolites) and biomarkers did not show significant differences between the groups of patients with and without complications in our study. Accordingly, the prognostic value of these parameters was not confirmed in this cohort. Although the EuroScore2 scale is a widely accepted tool for pre-operative risk assessment based on clinical and demographic data risks [37], a direct comparison with other models was not necessary or appropriate in this study. This is because the studied approaches assess different aspects of risk. However, our results indicate the need for further studies with a larger sample of patients to more accurately assess the prognostic value of the microbial metabolites and biomarkers.

The SOFA scale showed the best results in univariate models with the best specificity (ROC-AUC = 0.79 and sensitivity/specificity of 0.67/0.84), despite the fact that it is usually used for mortality prediction and it may have different predictive ability for various pathologic conditions [38]. We also examined the model on lactate—ROC-AUC = 0.62 and sensitivity/specificity of 0.74/0.61, and it may be compared with data on the predictive ability of this biomarker with those in other conditions, despite the fact that lactate is not used as a specific biomarker for cardiac surgery [39,40]. 

In our previous study, we received ROC-AUC = 0.71 and sensitivity/specificity of 0.81/0.56 for Σ3AMM and ROC-AUC = 0.69, and sensitivity/specificity of 0.79/0.47 for p-HPhLA for the prediction of all types of post-operative complications after aortic prosthetics [17]. In the current study, two univariate models using ΔΣ3AMM and Δp-HPhLA for the prediction of complications were built, and demonstrated predictive ability with ROC-AUC = 0.70 and sensitivity/specificity of 0.59/0.79 for ΔΣ3AMM, and ROC-AUC = 0.58, with the best sensitivity among all models—0.85 and poor specificity—0.50 for Δp-HPhLA.

Our pilot single-center study has a number of limitations. Firstly, the number of patients was relatively low, which meant we could not evaluate the contribution of sex, age, comorbidity, and off-pump surgeries on the statistics and quality of predictive models. Secondly, we considered all types of complication, despite the fact that different types of complication have different developmental pathophysiology. However, we supposed that the microbiota disruption, indirectly assessed by the level of microbial metabolites, could affect the development of all types of complication. Also, there were data on some serum samples in the latest days (the third and sixth) after surgery that were not analyzed. The short follow-up period of the study restricted the ability to assess long-term outcomes and complications. A longer follow-up would provide a more comprehensive understanding of the predictive value of the proposed integrated approach. However, making therapeutic decisions at the earliest stages, namely before surgery or in the early post-operative period, is most important in cardiac surgery for the successful management of patients in the post-operative period.

In cardiac surgery patients who developed infectious complications in the post-operative period, statistically significant changes in the taxonomic composition of the microbiota were detected, even before surgery, compared to patients without complications, while there were no differences in routine clinical and specific biochemical markers [18]. This is a potential target for reducing the risks of infectious complications. In this work, we assessed only a part of the clinical parameters, microbiota metabolites, and biomarkers, and perhaps expanding their range in the future will allow us to identify complications specific to the development, even before surgery, which will make diagnosis faster and more accessible. 

## 5. Conclusions

Using levels of certain metabolites and biomarkers circulating in the blood, in combination with clinical data, can help improve the predictive ability of diagnostic algorithms. It is essential to explore methods to regulate microbiota metabolism to improve surgical outcomes in the future by finding alternatives to compensate for its functions. For these purposes, it is important to organize and conduct multi-center studies in which it would be possible to study various approaches to pre-operative influences on the microbiota of patients in order to reduce the incidence of any type of post-operative complication in cardiac surgery.

## Figures and Tables

**Table 1:** Medical and demographic characteristics, intraoperative parameters, and serum concentrations of metabolites and biomarkers before (point 0) and within a day after the surgery (point 1) in all patients (*n* = 62), uncomplicated (*n* = 36), and complicated (*n* = 26) groups of patients. The statistically significant differences between uncomplicated and complicated groups of patients are highlighted in bold (*p* < 0.05).

Parameter	Reference Values/Concentrations of Metabolites in Donors	All Patients (*n* = 62)	Uncomplicated Group (*n* = 36)	Complicated Group (*n* = 26)	Uncomp. vs. Comp., *p*-Value	All Patients vs. Donors, *p*-Value
Age, years	-	62 (57, 67)	61 (57, 67)	63 (56, 67)	0.62	-
Sex (males), *n*, %	-	44, 71.0%	13, 36.1%	5, 19.2%	0.15 *	-
Diabetes Mellitus, *n*, %	-	10, 16.1%	6, 16.7%	4, 15.4%	0.72 *	-
EF (%)	-	60 (49, 63)	58 (48, 63)	60 (51, 66)	0.33	-
EuroScore 2	-	0.91 (0.75, 1.16)	0.94 (0.78, 1.08)	0.90 (0.71, 1.35)	0.84	-
Hemoglobin, g/L	110–160	114.5 (107.0, 125.5)	113.0 (107.0, 124.5)	118.0 (103.3, 125.5)	0.89	ns
Leucocytes, 10^9^	4–9	16.10 (13.50, 19.87)	16.20 (13.65, 18.54)	15.98 (12.56, 20.62)	0.85	**
Vasopressors > 6 h	-	45, 72.6%	22, 61.1%	23, 88.5%	0.02 *	-
Mechanic ventilation > 12 h	-	9, 14.5%	0, 0%	9, 34, 6%	0.001 *	-
Lactate, mmol/L	<2	5.17 (3.54, 7.08)	4.84 (3.46, 6.08)	5.85 (3.94, 7.94)	0.07	**
Infusion volume, mL	-	5928 (5175, 6897)	5560 (4822, 6425)	6825 (5888, 7600)	**0.001**	-
On-pump time, h	-	85.50 (76.25, 108.75)	78.50 (67.50, 98.50)	102.00 (85.00, 129.75)	**0.001**	-
pH max	7.35–7.45	7.47 (7.45, 7.50)	7.47 (7.44, 7.49)	7.49 (7.45, 7.50)	0.21	ns
pH min	7.35–7.45	7.33 (7.29, 7.36)	7.33 (7.29, 7.36)	7.33 (7.29, 7.35)	0.40	ns
SOFA on the 1st day	-	4 (3, 6)	4 (2, 4)	6 (4, 6)	**0.001**	-
Number of erythrocytes transfusions	-	1 (0, 2)	1 (0, 2)	2 (1, 3)	**0.03**	-
Blood loss on the 1st day, mL	-	1275 (1050, 1400)	1200 (1050, 1300)	1350 (1075, 1487)	**0.04**	-
Before surgery, point 0						
BA_0, µmol/L	<0.5 (<0.5, <0.5)	1.8 (1.3, 2.4)	1.7 (1.3, 2.3)	2.1 (1.5, 2.6)	0.13	**0.001**
PhAA_0, µmol/L	<0.5 (<0.5, 0.6)	0.9 (0.6, 1.2)	1.0 (0.6, 1.5)	0.9 (0.6, 1.0)	0.24	**0.001**
PhPA_0, µmol/L	<0.5 (<0.5, 0.5)	<0.5 (<0.5, <0.5)	<0.5 (<0.5, 0.5)	<0.5 (<0.5, <0.5)	0.33	0.75
PhLA_0, µmol/L	<0.5 (<0.5, <0.5)	<0.5 (<0.5, <0.5)	<0.5 (<0.5, <0.5)	<0.5 (<0.5, <0.5)	0.85	0.79
p-HBA_0, µmol/L	<0.5 (<0.5, <0.5)	<0.5 (<0.5, <0.5)	<0.5 (<0.5, <0.5)	<0.5 (<0.5, <0.5)	0.19	**0.03**
p-HPhAA_0, µmol/L	<0.5 (<0.5, <0.5)	0.7 (0.5, 1.0)	0.7 (0.5, 1.1)	0.6 (0.5, 0.9)	0.45	**0.001**
p-HPhPA_0, µmol/L	<0.5 (<0.5, <0.5)	<0.5 (<0.5, <0.5)	<0.5 (<0.5, <0.5)	<0.5 (<0.5, <0.5)	1.00	1
HVA_0, µmol/L	<0.5 (<0.5, <0.5)	<0.5 (<0.5, <0.5)	<0.5 (<0.5, <0.5)	<0.5 (<0.5, <0.5)	0.74	0.13
p-HPhLA_0, µmol/L	1.2 (1.0, 1.6)	1.2 (1.0, 1.4)	1.2 (1.0, 1.4)	1.2 (1.1, 1.4)	0.72	0.37
Σ3AMM_0, µmol/L	1.9 (1.5, 2.2)	2.1 (1.8, 2.7)	2.1 (1.8, 2.8)	2.0 (1.7, 2.5)	0.55	**0.04**
SA_0, µmol/L	4.8 (4.4, 6.0)	20.2 (17.0, 25.2)	19.9 (16.9, 25.8)	20.7 (17.6, 24.0)	0.86	**0.001**
FA_0, µmol/L	1.3 (1.1, 1.5)	1.7 (1.5, 2.1)	1.7 (1.4, 2.1)	1.7 (1.5, 2.4)	0.93	**0.001**
IL6_0, pg/mL	<7	9.0 (6.8, 13.6)	9.5 (7.0, 13.6)	8.7 (6.2, 14.2)	0.75	ns
PCT_0, ng/mL	<0.5	<0.5 (<0.5, <0.5)	<0.5 (<0.5, <0.5)	<0.5 (<0.5, <0.5)	0.59	ns
NT-proBNP_0, pg/mL	<300	564.8 (216.0, 883.0)	478.3 (213.7, 790.3)	625.2 (239.8, 883.0)	0.72	ns
S100_0, pg/mL	<0.5	<0.5 (<0.5, <0.5)	<0.5 (<0.5, <0.5)	<0.5 (<0.5, <0.5)	0.27	ns
HST-T_0, pg/mL	<0.5	24.2 (16.2, 40.3)	29.2 (16.5, 45.3)	22.5 (15.6, 30.4)	0.16	ns
A day after surgery, point 1						
BA_1, µmol/L	<0.5 (<0.5, <0.5)	1.4 (0.9, 2.8)	1.2 (0.9, 2.1)	1.6 (1.1, 3.1)	0.15	**0.001**
PhAA_1, µmol/L	<0.5 (<0.5, 0.60)	<0.5 (<0.5, 0.5)	<0.5 (<0.5, 0.5)	<0.5 (<0.5, <0.5)	0.99	**0.001**
PhPA_1, µmol/L	<0.5 (<0.5, 0.52 < 0)	5 (<0.5, <0.5)	<0.5 (<0.5, <0.5)	<0.5 (<0.5, <0.5)	1.00	**0.001**
PhLA_1, µmol/L	<0.5 (<0.5, <0.5)	<0.5 (<0.5, <0.5)	<0.5 (<0.5, <0.5)	<0.5 (<0.5, <0.5)	0.96	**0.001**
p-HBA_1, µmol/L	<0.5 (<0.5, <0.5)	<0.5 (<0.5, <0.5)	<0.5 (<0.5, <0.5)	<0.5 (<0.5, <0.5)	0.77	**0.03**
p-HPhAA_1, µmol/L	<0.5 (<0.5, <0.5)	<0.5 (<0.5, <0.5)	<0.5 (<0.5, <0.5)	<0.5 (<0.5, <0.5)	0.49	**0.001**
p-HPhPA_1, µmol/L	<0.5 (<0.5, <0.5)	<0.5 (<0.5, <0.5)	<0.5 (<0.5, <0.5)	<0.5 (<0.5, <0.5)	1.00	**0.001**
HVA_1, µmol/L	<0.5 (<0.5, <0.5)	1.6 (0.7, 3.0)	1.1 (<0.5, 3.0)	2.3 (1.1, 3.3)	0.06	**0.001**
p-HPhLA_1, µmol/L	1.3 (1.0, 1.6)	1.6 (1.3, 2.4)	1.6 (1.3, 2.3)	1.6 (1.3, 2.4)	0.56	**0.001**
Σ3AMM_1, µmol/L	1.9 (1.5, 2.2)	2.2 (1.8, 3.3)	2.4 (1.7, 3.3)	2.2 (1.8, 3.2)	0.98	**0.001**
SA_1, µmol/L	4.8 (4.4, 5.9)	16.2 (12.7, 21.7)	15.8 (12.2, 20.0)	16.9 (14.3, 23.3)	0.34	**0.001**
FA_1, µmol/L	1.3 (1.1, 1.5)	2.2 (1.5, 3.8)	2.2 (1.5, 4.5)	2.1 (1.8, 3.4)	0.91	**0.001**
IL6_1, pg/mL	<7	67.6 (38.0, 101.1)	58.7 (34.8, 102.8)	76.4 (43.7, 100.0)	0.64	**
PCT_1, ng/mL	<0.5	3.3 (0.7, 8.8)	1.5 (<0.5, 7.3)	4.2 (1.1, 16.8)	0.06	**
NT-proBNP_1, pg/mL	<300	981.9 (608.9, 1409.3)	964.1 (468.8, 1329.8)	1181.5 (817.8, 1878.8)	0.13	**
S100_1, pg/mL	<0.5	<0.5 (<0.5, <0.5)	<0.5 (<0.5, <0.5)	<0.5 (<0.5, <0.5)	0.81	ns
HST-T_1, pg/mL	<0.5	356.7 (202.4, 614.0)	261.9 (194.2, 455.0)	575.5 (216.9, 745.5)	**0.03**	**
Differences between point 1 and point 0 (Δ)						
ΔBA, µmol/L	-	−0.3 (−2.5, 1.9)	−0.4 (−2.0, 1.3)	−0.2 (−3.0, 2.5)	0.89	-
ΔPhAA, µmol/L	-	−0.8 (−1.4, −0.2)	−0.8 (−1.5, −0.1)	−0.7 (−1.2, −0.3)	0.32	-
ΔPhPA, µmol/L	-	0.0 (−0.2, 0.2)	0.0 (−0.3, 0.3)	0.0 (−0.1, 0.1)	0.37	-
ΔPhLA, µmol/L	-	0.0 (−0.2, 0.2)	0.0 (−0.3, 0.3)	0.0 (−0.2, 0.2)	0.65	-
Δp-HBA, µmol/L	-	0.0 (−0.8, 0.8)	0.0 (−0.9, 0.9)	0.0 (−0.5, 0.5)	0.28	-
Δp-HPhAA, µmol/L	-	−0.6 (−1.8, 0.7)	−0.7 (−2.1, 0.7)	−0.5 (−1.4, 0.3)	0.22	-
Δp-HPhPA, µmol/L	-	0.0 (0.0, 0.0)	0.0 (0.0, 0.0)	0.0 (0.0, 0.0)	0.99	-
ΔHVA, µmol/L	-	1.7 (−2.4, 5.8)	1.1 (−2.4, 4.7)	2.3 (−2.2, 6.7)	0.08	-
Δp-HPhLA, µmol/L	-	0.5 (−0.3, 1.3)	0.4 (−0.2, 1.1)	0.6 (−0.3, 1.5)	0.20	-
ΔΣ3AMM, µmol/L	-	0.3 (−1.2, 1.9)	0.0 (−1.5, 1.5)	0.7 (−0.8, 2.2)	**0.03**	-
ΔSA, µmol/L	-	−4.4 (−16.3, 7.6)	−4.9 (−15.8, 5.9)	−2.3 (−14.9, 10.4)	0.29	-
ΔFA, µmol/L	-	0.5 (−8.7, 9.7)	0.4 (−11.0, 11.7)	0.5 (−4.2, 5.3)	0.96	-
ΔIL6, pg/mL	-	60.8 (−28.6, 150.1)	51.6 (−31.2, 134.4)	69.3 (−28.4, 167.0)	0.97	-
ΔPCT, ng/mL	-	3.6 (−11.6, 18.8)	1.5 (−4.2, 7.1)	4.9 (−16.2, 25.9)	**0.01**	-
ΔNT-proBNP, pg/mL	-	493.6 (−1878.4, 2865.7)	264.4 (−871.4, 1400.1)	720.6 (−2547.1, 3988.3)	**0.01**	-
ΔS100, pg/mL	-	0.0 (−0.3, 0.3)	0.0 (−0.2, 0.2)	0.0 (−0.4, 0.5)	0.67	-
ΔHST-T, pg/mL	-	390.7 (47.8, 733.6)	247.0 (−106.4, 600.4)	569.5 (270.4, 868.6)	**0.01**	-

*—Chi-square test; **—reference values out of 95% CI, the differences are significant; ns—not significant differences.

**Table 2:** Characteristics of multi- and univariate predictive models for the post-operative cardiac complications.

Models	ROC-AUC, 95% CI	Sensitivity, 95% CI	Specificity, 95% CI
Multivariate models			
All data	0.85 (0.78, 0.92)	0.81 (0.77, 0.85)	0.79 (0.75, 0.83)
Clinical data	0.80 (0.71, 0.89)	0.66 (0.63, 0.69)	0.78 (0.72, 0.84)
Metabolites and biomarkers	0.71 (0.56, 0.86)	0.72 (0.69, 0.75)	0.47 (0.43, 0.51)
Univariate models			
SOFA	0.79 (0.73, 0.85)	0.67 (0.62, 0.72)	0.84 (0.75, 0.93)
ΔΣ3AMM	0.70 (0.62, 0.78)	0.59 (0.55, 0.73)	0.79 (0.75, 0.83)
Lactate	0.62 (0.42, 0.82)	0.74 (0.66, 0.82)	0.61 (0.56, 0.66)
Δp-HPhLA	0.58 (0.47, 0.69)	0.85 (0.80, 0.90)	0.48 (0.41, 0.55)
EuroScore2	0.54 (0.38, 0.70)	0.49 (0.3, 0.55)	0.63 (0.59, 0.67)

## Data Availability

The original contributions presented in the study are included in the article, further inquiries can be directed to the corresponding author.
